# Efficiency of glass ionomer sealant application in reducing hypersensitivity in MIH-molars in schoolchildren immediately and after 12 weeks

**DOI:** 10.1007/s40368-024-00988-2

**Published:** 2025-01-17

**Authors:** R. Karim, M. Baider, C. H. Splieth, J. Schmoeckel

**Affiliations:** Department of Paediatric Dentistry, University Medicine Greifswald, Walther-Rathenau-Straße 42a, Greifswald, Germany

**Keywords:** Hypersensitivity, Molar incisor hypomineralisation, Glass-ionomer cement, Schiff score air sensitivity scale, Wong-baker faces Scale

## Abstract

**Aim:**

This prospective clinical study aimed to clinically investigate the efficiency of (GIC) glass-ionomer cement application (Ionostar Plus + Easy Glaze, VOCO) in reducing hypersensitivity in permanent molars affected by molar incisor hypomineralisation when assessed immediately (15 min) and 12 weeks after its application.

**Materials and Methods:**

Children with at least one hypersensitive MIH-affected permanent molar (MIH-TNI-3 or 4). The pre-treatment status was evaluated and only included if they did not receive a tooth-specific in-office desensitizing treatment within one month. Clinical pain assessments were performed using the schiff score air sensitivity scale (SCASS) and Wong-baker faces Scale (WBFS).

**Results:**

This study involved 25 participants (mean age 8.6 ± 1.85 years) with 43 hypersensitive MIH-molars of which about half were SCASS 3 (*n* = 24, 55.8%) and SCASS 2 (*n* = 19, 44.2%). Regarding hypersensitivity, the reported mean SCASS score reduced significantly from 2.56 (± 0.50) at baseline to 1.14 (± 0.96) after 15 min of GIC sealant application and even further to 0.71 (± 0.89) after 12 weeks (*P* < 0.001, Paired *t* test). Similarly, reported WBFS scores also significantly reduced from 5.81 (± 2.50; Range = 2–10) at baseline to 2.88 (± 2.31; Range = 0–10) after 15 min and to 2.95 (± 2.17) after 12 weeks, respectively (*P* < 0.001, Paired *t* test). The mean reduction in SCASS scores was 1.3 (± 0.6) and 1.4 (± 1.0) for baseline SCASS 2 vs. 3, respectively.

**Conclusion:**

GIC coverage is effective in providing clear instant relief from hypersensitivity in MIH molars in schoolchildren, which improves even further over a period of 12 weeks.

**Supplementary Information:**

The online version contains supplementary material available at 10.1007/s40368-024-00988-2.

## Introduction

Molar incisor hypomineralisation (MIH) is a qualitative developmental defect of unknown origin with a worldwide prevalence of 14.2% that affects one or more first permanent molars with or without incisors involvement (Zhao et al. 2018). Clinical features of MIH involve a demarcated opacity with chromatic changes that vary from white/cream to yellow/brownish, which can be seen as an alteration in enamel translucency (Weerheijm 2003). In addition, the condition is associated with clinical complications such as enamel loss, increased risk of caries development, post-eruptive breakdown due to masticatory efforts and mostly severe hypersensitivity resulting in pain and discomfort (Lygidakis 2010).

Hypersensitivity is considered as one major symptom in children with MIH with a prevalence of 23% (Raposo et al. 2019). The reason for this hypersensitivity is still not clearly understood in detail. However, the affected enamel is characterized by an increased porosity which leads to reduced thermal isolation properties and altered thermal conductivity (Fagrell et al. 2010). This increased hypersensitivity leads to deterioration of oral hygiene with increased plaque accumulation resulting in increased susceptibility to caries formation (Americano et al., 2017). MIH-affected teeth are nearly 10 times more susceptible to caries development and exhibit difficulty in dental anaesthesia (Jalevik & Klingberg, 2002). These clinical complications of MIH have functional, aesthetic and social effects resulting in reduced oral health-related quality of life (OHRQoL) of children (Awwad et al. 2023).

The management of MIH teeth varies based on severity. A wide range of treatment options are currently available ranging from caries and enamel breakdown prevention, management of hypersensitivity, restorative treatment, and extraction with or without combined orthodontic treatment (Lygidakis et al. 2022; Bekes et al. 2023). Preventive management involves thorough oral hygiene instruction, brushing with fluoride-containing toothpaste as well as the application of other topical fluoride varnish (e.g.22.600 ppm fluoride) combined with frequent recall visits (Baroni & Marchionni 2011).

Regarding hypersensitivity several products are suggested for remineralisation and desensitisation purposes. Such as casein phosphopeptide-amorphous calcium phosphate (CPP-ACP), which showed a superior effect in the reduction of tooth hypersensitivity compared to usual fluoride toothpastes (Pasini et al., 2018). In addition, similar results were reported with arginine-containing products, which reported a significant reduction in the degree of hypersensitivity in MIH molars after a period of 3 months (Bekes et al. 2017). Whereas, silver diamine fluoride (SDF) is also looked at as non-invasive desensitising agent, which acts to block dentinal tubules by producing fluorohydroxyapatite and increasing mineral density and hardness resulting in relief of tooth hypersensitivity but with the draw-back of potential black discolorations (Al-Nerabieah et al., 2024).

The preventive approach through sealant application is known as an acceptable, widely available and feasible for prevention or control of caries on occlusal surface, which enhance tissue remineralisation, inhibit the biofilm formation and provide a cleansable surface (Ahovuo-Saloranta et al. 2017). Furthermore, sealed occlusal surfaces in children showed a significant reduction in caries progression compared to unsealed occlusal surfaces (Llodra et al. 1993). Children affected with MIH are more frequently anxious. This may make restorative procedures challenging. Hence the use of simplified sealant application avoiding adhesively bonded sealants, which involves the use of conventional acid etching and water spray and drying making the procedure technique sensitive, and hence increasing patients discomfort in hypersensitive teeth.

Generally spoken, current guidelines such as the “Würzburg concept 2.0” suggest the application of a sealant for MIH teeth with and without breakdown as an initial therapy to reduce pain (Bekes et al. 2023). This is supported by the current practice guidance of European Academy of Paediatric Dentistry, in which GIC sealant is defined as an atraumatic restoration in children, who lack cooperation for invasive treatment requiring local anaesthetics (Lygidakis et al. 2022). Nevertheless, few clinical studies have been published regarding the immediate and mid-term effect of GIC sealant on the reduction of hypersensitivity in MIH molars with and without enamel breakdown which may not be used to influence caries risk but to reduce hypersensitivity via an easy tool. Therefore, the aim of this study is to prospectively investigate the clinical efficiency of (GIC) glass-ionomer cement application (Ionostar Plus + Easy Glaze, VOCO) in reducing hypersensitivity in permanent molars affected by molar incisor hypomineralisation when assessed immediately (15 min) and 3 months after its application.

## Material and methods

**Fig. 1:**
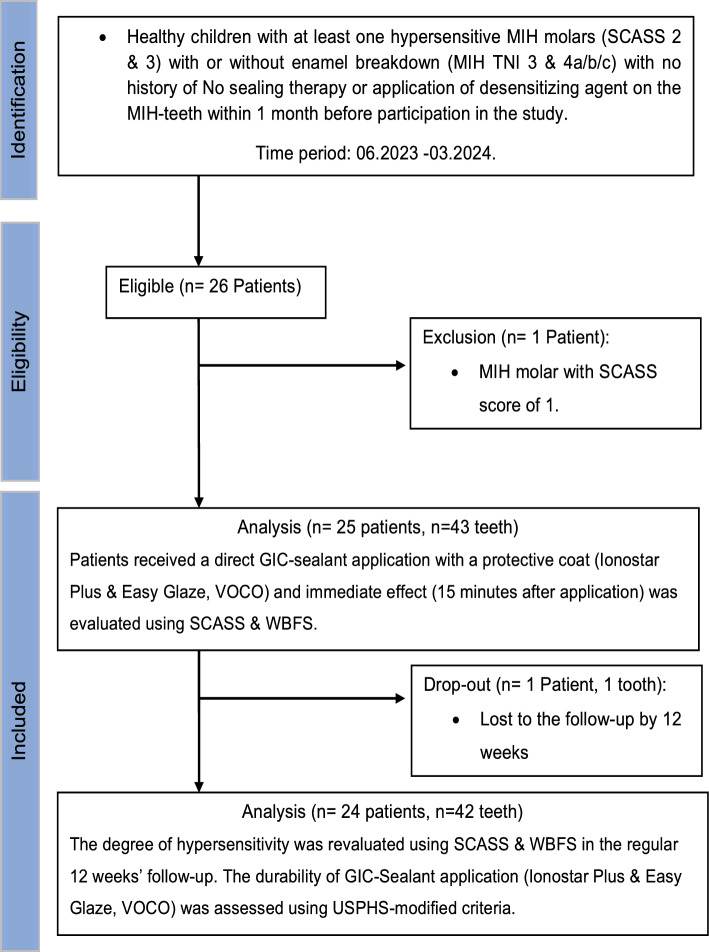
Flow-chart of the study design

This prospective interventional clinical study was approved by the Ethics Committee (BB 047 /23) of the University medicine Greifswald, Germany and was conducted in strict adherence to the STROBE checklist (appendix 1). The study protocol was registered on ClinicalTrials (NCT05945381). All caregivers were asked to sign an informed consent after thorough explanation of the study procedures and outcomes of treatment. Children would have been excluded from the study when their caregivers had refused to give informed consent. As shown in the flow-chart of the study representing the outline of the study design all eligible patients gave consent (Fig. 1) but one patient was excluded as not all inclusion criteria were fulfilled.

### Selection of the participants

The participants of this study were either new patients to the paediatric dentistry department of the University Dental Hospital (Greifswald) with newly erupted hypersensitive MIH molars or recall patients with persisting hypersensitivity. These children usually had a history of desensitising treatment which was recorded based on their dental records. For the diagnosis of MIH teeth, the criteria defined by the Würzburg concept based on the MIH treatment need index (MIH-TNI) was used (Steffen et al., 2017).

The inclusion criteria of the study were as follows:

- Self-reported hypersensitive MIH molar with and/or without enamel breakdown (MIH-TNI 3 & 4a/b/c)
- Hypersensitive MIH affected molars with a score of 2 or 3 on the Schiff cold air sensitivity score (SCASS; Schiff et al. 2009)
- Children & adolescents without systematic health diseases
- No sealing therapy or application of desensitizing agent on the MIH teeth within 1 month before participation in the study
- Agreement to voluntary participation in the study (informed consent)

Whereas the exclusion criteria involved:

- Patients with acute pain/ patients with signs or symptoms to be treated
- Patients with systemic disease that require special attention during their dental treatment
- Parents / children who refused to participate in the study.
- MIH teeth with signs of irreversible pulpitis
- Allergy against any ingredients of the study material

### Sample size calculation

Sample size calculation was performed based on findings from a previous study, showing a minimum significant difference of 1 in SCASS scores (Bekes et al. 2017). The probability of the study detecting a treatment difference was assumed to be 80% at a two-sided 0.05 significance level and resulted in a required enrolment of at least 19 participants with at least one affected MIH molar. To reduce the risk of drop-out (of approximately 25%) for the 12 weeks’ follow-up, an increase of 25% lead to a final required baseline sample of 25 participants.

### Assessment tools

The intra-oral examination of the potential participant for inclusion, baseline assessment, immediate effect (15 min after application) and 12 weeks’ reassessment were performed by two trained and calibrated study dentists (RK & MB). In each case, the grade of the hypersensitivity of the MIH teeth was performed in response to the air-blast hypersensitivity test via schiff cold air sensitivity score (SCASS). The air blast was applied perpendicularly on the occlusal surface of the tooth for 1 s at a distance of 1 cm. The adjacent teeth were shielded with the finger of the examiner, the same shielding technique was used in previous studies to assess MIH hypersensitivity in schoolchildren (Bekes et al. 2022, 2017). The patient’s response to the air blast was recorded according to the SCASS score as shown below in Table 1 which is considered the more objective parameter (Schiff et al. 2009). Whereas the subjective pain intensity of the participant to the air-blast test was recorded with the Wong baker faces scale (WBFS) resembling the range “0 = no hurt” to “10 = hurts worst” (Wong & Baker, 1998).

**Table 1:** Schiff Cold Air Sensitivity Score (SCASS; Schiff et al. 2009**)**

Grade	Description
0	Subject does not respond to the stimulus
1	Subject does not respond to the stimulus, but considers stimulus to be painful
2	Subject responds to air stimulus and moves from the stimulus
3	Subject responds to air stimulus, moves from the stimulus, and requests immediate discontinuation of the stimulus

Study dentists and patient blinding were not possible due to the intervention procedures of the study. However, to reduce the risk of bias in recording the primary outcome, the study adheres to the ROBINS-I tool for non-randomised -prospective clinical study (Sterne et al. 2016). Thus, the reassessment of the degree of hypersensitivity via SCASS following direct application of GIC sealant application (15 min) and in 12 weeks was performed not only by two principal investigators (RK & MB) but also through an additional independent trained assessor (3 different paediatric dentists involved). In the rare case of a different assessment the SCASS score included for analysis was chosen by discussion and agreement between the examiners right after clinical evaluation.

The modified United States Public Health Service (USPHS) clinical rating system was used for clinical evaluation of the GIC-sealants (Ionostar Plus & Easy Glaze, VOCO) retention rate, marginal integrity, discoloration surface texture (optic and tactile) and presence of caries lesion at the follow-up visit scheduled after 12 weeks (Dukic et al., 2007).

### Intervention / procedure

In each patient, the hypersensitive MIH tooth/teeth was/were sealed with a fast-setting radiopaque bulk-fill glass-ionomer filling material (Ionostar Plus, VOCO) followed by an application of a nano-filled, light-curing coating (Easy Glaze, VOCO) with a natural fluorescence for surface sealing.

Before sealing, the hypersensitive MIH teeth were cleaned with prophylactic paste for tooth-cleaning (rophy paste, Henry Schein) using a bristle brush. The teeth were then isolated with cotton rolls and the glass-ionomer cement (Ionostar Plus, VOCO) was then applied after mixing the capsule for 10 s. Subsequently, the sealants were covered with a protective coat (Easy Glaze, VOCO) and cured for 30 s based on manufacturer instructions. Occlusion control was performed using articulating paper and adjustment of the GIC sealant were only done if it was necessary. The study procedures were done without the administration of local anaesthesia (LA).

Finally, the patients were instructed to brush daily with fluoride containing toothpaste and use weekly a fluoride gel (12.500 ppm fluoride) as generally suggested for patients with high caries risk in the department (AAPD, 2023).

### Data analysis

The data were initially summarised descriptively, and the mean value and standard deviation were calculated. The mean SCASS and WBFS between different time periods were compared using *t* test. Whereas, the difference between measurements at different time points was calculated using the Mann–Whitney *U* Test. The inter-examiner reliability was calculated using Cohen’s Kappa test. The inter-examiner reliability for reassessing the degree of hypersensitivity via SCASS scores directly after the application (15 min) and in 3 months’ period via the study dentists and independent examiner was 0.80. In case of disagreement, a consensus scoring was made. All statistical tests were performed with the Excel 2016 program (Excel, Microsoft). In this study, a *P* > 0.05 was considered to be statistically significant.

## Results

After screening and assessing the eligibility to participate in the study, 25 patients (mean age 8.6 ± 1.85, 56% females) with a total number of 43 teeth were included in this prospective study. The patients had a mean dmft/DMFT of 2.46 (± 3.06)/0.80 (± 0.87). An average number of ~ 1.7 teeth with high intensity of hypersensitivity (SCASS ≥ 2) were included in MIH-affected patients as shown in Table 1.

Table 2 shows the general baseline characteristics on tooth level. More than half of the hypersensitive MIH molars (*n* = 26, 60.5%) were upper right (16) and lower left (46) permanent molars and a SCASS score of 3 (*n* = 24, 55.8%). A total number of 30 teeth (69.8%) had entirely no history of prior in-office desensitising treatment, the other ones (*n* = 13, 31.2%) had but more than 1 month before study enrolment. Previous treatments included e.g. silver diamine fluoride, glass-ionomer cement or composite fillings (Table 2).

**Table 2:** General baseline characteristic of the study sample (Patient level; n = 25)

Patient variable	Category	
Age (in years)	Mean(± SD)	8.6 ± 1.85
	Min–Max	6.2–11.7
Sex	Males	11 (44.0%)
	Females	14 (56.0%)
Dmft	Mean(± SD)	2.46 (± 3.06)
	Min–Max	0.00–9.00
DMFT	Mean(± SD)	0.80 (± 0.87)
	Min–Max	0.00–3.00
Mean follow-up time (for scheduled 12 weeks follow-up; Weeks)	Mean(± SD)	16.3 (± 5.0)
Number of hypersensitive MIH molars per patient receiving GIC application	Mean(± SD)	1.72 (± 1.02)
	Min–Max	1.00–4.00

*SD* Standard deviation, *Min* Minimum, *Max* Maximum

The intensity of the hypersensitivity was evaluated via SCASS and WBFS at different time points (baseline, 15 min and 12 weeks post-application) as shown in Table 3 and 4. According to the study results, a statistical significant reduction in the degree of hypersensitivity was observed in all 25 patients immediately (15 min post application) and 12 weeks measured with SCASS as well as WBFS (*P* < *0.001, paired t test).* The mean SCASS scores decreased significantly immediately following GIC application (Ionostar Plus & Easy Glaze, VOCO) to 1.14 (± 0.96) and to 0.71 (± 0.89) after 12 weeks (Table 3 and 4). Moreover, the mean WBFS scores also significantly decreased to 3.00 (± 2.28) after 15 min and basically stayed constant with a mean value of 2.95 (± 2.17) when the child was asked after air stimulus in the 12 week follow-up (Table5).

**Table 3:** General baseline characteristic of the study sample (tooth level; *n* = 43)

Category	Tooth distribution (included hypersensitive MIH molar)					Total *N* (%)
Criteria	#16	#26	#36	#46	#17	
MIH-TNI score						
3	8 (66.6%)	4 (57.1%)	2 (22.2%)	5 (35.7%)	–	19 (44.1%)
4a	4 (44.4%)	2 (28.6%)	5 (55.6%)	5 (35.7%)	–	16 (37.2%)
4b	–	1 (14.3%)	2 (22.2%)	4 (28.6%)	1 (100%)	8 (18.6%)
4c	–	–	–	–	–	–
Total	12 (27.9%)	7 (16.3%)	9 (20.1%)	14 (33.5%)	1 (2.3%)	43 (100%)
Baseline SCASS						
2	6 (50.0%)	2 (28.6%)	2 (22.2%)	9 (64.3%)	–	19 (44.2%)
3	6 (50.0%)	5 (71.4%)	7 (77.8%)	5 (35.7%)	1 (100%)	24 (55.8%)
Total	12 (27.9%)	7 (16.3%)	9 (20.1%)	14 (32.5%)	1 (2.3%)	43 (100%)
Prior desensitising treatment (> 1 month before study enrolment)						
N/A	9 (75.0%)	6 (85.7%)	5 (55.6%)	9 (64.2%)	1 (100%)	30 (69.8%)
SDF	1 (8.3%)	–	–	1 (7.1%)	–	2 (4.7%)
Flg	–	–	1 (11.1%)	1 (7.1%)	–	2 (4.7%)
GIC	–	–	2 (22.2%)	3 (21.4%)	–	5 (11.6%)
GIC + Flg	1 (8.3%)	–	1 (11.1%)	-	–	2 (4.7%)
GIC + SDF	1 (8.4%)	1 (14.2%)	–	–	–	2 (4.7%)
Total	12 (27.9%)	7 (16.3%)	9 (20.1%)	14 (32.5%)	1 (2.3%)	43 (100%)

*SDF* Silver-diamine fluoride, *GIC* Glass-ionomer cement, *Flg*: Composite filling, *N/A* Not applicable

**Table 4:** Comparison of SCASS scores at baseline and after 15 min & 12 weeks post application of GIC on hypersensitive MIH molars (tooth level; *n* = 43; Drop-out, *n* = 1)

SCASS score	Baseline	15 min	12 weeks	Paired *t* test
	*N* (%)			*P* value
0	–	13 (30.2%)	22 (52.4%)	*P* < 0.001*
1	–	15 (34.9%)	12 (28.5%)	
2	19 (44.2%)	11 (25.6%)	6 (14.3%)	
3	24 (55.8%)	4 (9.3%)	2 (4.7%)	
Mean (SD)	2.56 (± 0.50)	1.14 (± 0.96)	**0.71 (± 0.89)**	
Median (IQR)	3.00 (2.00, 3.00)	1.00 (1.00, 2.00)	**0.00 (0.00, 1.00)**	
Difference (15 min–baseline)	Mean (SD) = − 1.42 (0.46)			
	Median (IQR) = − 2.00 (− 1.00, − 1.00)			
Difference (12 weeks–baseline)	Mean (SD) = − 1.85 (0.39)			*P* < 0.001*
	Median (IQR) = − 3.00 (− 2.00, − 2.00)			
Difference (15 min–12 weeks)	Mean (SD) = − 0.44 (0.09)			4 < 0.01*
	Median (IQR) = − 1.00 (− 1.00, − 1.00)			

*SCASS* Schiff cold air sensitivity test, *SD* Standard deviation, *IQR* Interquartile range, Paired t test was used. *statistically significant at *p* value < 0.05

**Table 5:** Comparison of WBFS scores at baseline and after 15 min & 12 weeks post application of GIC on hypersensitive MIH molars (tooth level; *n* = 43; Drop-out, *n* = 1)

WBFS score	Baseline	15 min	12 weeks	Paired *t* test
	*N* (%)			*P* value
≤ 4	15 (34.9%)	37 (86.2%)	35 (83.4%)	*P* < 0.01*
≥ 6	28 (65.10%)	6 (13.8%)	7 (16.6%)	
Mean (SD)	5.86 (± 2.50)	3.00 (± 2.28)	2.95 (± 2.17)	
Median (IQR)	6.00 (4.00, 8.00)	2.00 (2.00, 4.00)	4.00 (2.00, 4.00)	
Difference (15 min–baseline)	Mean (SD) = − 2.86 (0.22)			
	Median (IQR) = − 4.00 (− 2.00, − 4.00)			
Difference (12 weeks–baseline)	Mean (SD) = − 2.91 (0.33)			*P* < 0.01*
	Median (IQR) = − 2.00 (− 2.00, − 4.00)			
Difference (15 min–12 weeks)	Mean (SD) = − 0.05 (0.11)			*P* > 0.05
	Median (IQR) = 2.00 (0.00, 0.00)			

*WBFS* Wong-Baker Facial Scale, *SD* Standard deviation, *IQR* Interquartile range, Paired t test was used. *statistically significant at p value < 0.0

Furthermore, hypersensitive MIH molars with a baseline SCASS score of 2 & 3 showed a significant reduction in the hypersensitivity both immediately (15 min) and after 12 weeks after application of the GIC sealant (*P* = *0.01, Mann–Whitney U test*). According to the study results, no significant difference (P = 0.06**) in the extent of the immediate hypersensitivity relief (15 min after the application of GIC (Ionostar Plus + Easy Glaze, VOCO) between MIH molars of a baseline SCASS 2 vs. SCASS 3 (*P* = *0.06, t test) was achieved* (Table 6). However, the hypersensitivity of MIH molars with baseline SCASS score 2 was reduced significantly more than those of a baseline SCASS score 3 in the time period of 3 months (mean SCASS reduction ± SD: − 1.96 ± 0.41 vs. − 1.68 ± 0.41; Table 5).

**Table 6:** Comparison of SCASS scores after 15 min and 12 weeks post treatment with GIC differentiated by baseline SCASS score (2 vs. 3) molars (tooth level; *n* = 43; Drop-out, *n* = 1)

15 min post-treatment SCASS score	Baseline SCASS 2 (*n* = 19; 44.2%)	Baseline SCASS 3 (*n* = 24; 55.8%)	*P* value
	N (%)		
0	7 (36.8%)	6 (25.0%)	*P* = 0.01*
1	10 (52.6%)	5 (20.8%)	
2	2 (10.5%)	9 (37.5%)	
3	–	4 (16.7%)	
Mean (± SD)	0.74 (± 0.98)	1.46 (± 0.95)	
Median (IQR)	1.00 (0.00, 1.00)	1.00 (1.00, 2.00)	
Difference			
Mean (SD)	− 1.26 (± 0.48)	− 1.54 (± 0.46)	*P* = 0.06**
Median (IQR)	− 1.00 (− 2.00, − 1.00)	− 2.00 (− 2.00, − 1.00)	

12 weeks post-treatment SCASS score	Baseline SCASS 2 (*n* = 19; 44.2%)	Baseline SCASS 3 (*n* = 23; 55.8%)	*P* value
	N (%)		
0	13 (68.4%)	9 (39.1%)	*P* = 0.03*
1	6 (31.6%)	6 (26.1%)	
2	–	6 (26.1%)	
3	–	2 (8.7%)	
Mean (± SD)	0.32 (± 0.91)	1.04 (± 0.90)	
Median (IQR)	0.00 (0.00, 1.00)	1.00 (0.00, 1.00)	
Difference			
Mean (SD)	− 1.68 (± 0.41)	− 1.96 (± 0.41)	*P* = 0.03**
Median (IQR)	− 1.00 (− 2.00, − 1.00)	− 2.00 (− 2.00, − 1.00)	

*SCASS* Schiff cold air sensitivity test; *SD* Standard deviation, *IQR* Interquartile range, *MWU* ^*^Mann–Whitney U test was used. **t test. statistically significant at *p* value < 0.05

In addition, a similar reduction in the intensity of hypersensitivity was observed in hypersensitive MIH molars with and without a history of in-office desensitising pre-treatment after 15 min within the GIC application (*P* = *0.62, Mann–Whitney U test)* and in 12 week’s revaluation (*P* = *0.31, Mann–Whitney U test)* as shown in Table 7. Furthermore, there were no significant differences in mean SCASS reductions between both groups immediately after application for teeth without pre-treatment history (− 0.92 (± 0.73) vs. − 1.33 (± 0.22)*, P* = *0.11, t test*) and after 12 week’s follow-up for pre-treatment and without pretreatment (− 1.61 (± 0.38) vs. − 1.38 (± 0.55), P = 0.35, *t test*) as shown above in Table 7. Though in this study, hypersensitive MIH molars with a history of pre-treatment (*n* = 13) showed a statistically significant higher reduction in mean SCASS, when compared between immediate effect (15 min) and 12 weeks follow-up (− 0.69 (± 0.35) vs. − 0.05 (± 0.12) *P* < *0.01*, *t test*).

**Table 7:** Comparison of SCASS scores differentiated by prior treatment in MIH molars (tooth level; *n* = 43; Drop-out, *n* = 1)

		Pre-treatment (*n* = 13)	No pre-treatment (*n* = 30)	*P* value
		*N* (%)		
Baseline SCASS				
0		–	–	*P* = 0.07*
1		–	–	
2		9 (40.9%)	10 (54.8%)	
3		4 (59.1%)	20 (45.2%)	
Mean (± SD)		2.30 (± 0.48)	2.45 (± 0.51)	
Median (IQR)		3.00 (2.00, 3.00)	2.00 (2.00, 3.00)	
15 min SCASS				
0		4 (36.4%)	9 (12.9%)	*P* = 0.62*
1		4 (31.8%)	9 (12.9%)	
2		1 (18.2%)	9 (12.9%)	
3		4 (13.6%)	9 (12.9%)	
Mean (SD)		1.38 (± 1.21)	9 (12.9%)	
Median (IQR)		1.00 (0.00, 2.00)	1.00 (0.00, 2.00)	
12 weeks SCASS (Drop-out; n = 1)				
0		7 (53.8%)	11 (37.9%)	*P* = 0.31*
1		3 (23.1%)	9 (31.3%)	
2		3 (23.1%)	5 (17.2%)	
3		-	4 (13.7%)	
Mean (SD)		0.69 (± 0.86)	1.07 (± 1.06)	
Median (IQR)		1.00 (0.00, 1.00)	1.00 (0.00, 1.00)	
Difference Baseline—15 min				
Mean (SD)		− 0.92 (± 0.73)	− 1.33 (± 0.22)	*P* = 0.11**
Median (IQR)		− 2.00 (− 2.00, − 1.00)	− 1.00 (− 2.00, − 1.00)	
Difference Baseline—12 weeks				
Mean (SD)		− 1.61 (± 0.38)	− 1.38 (± 0.55)	*P* = 0.35**
Median (IQR)		− 2.00 (− 2.00, − 2.00)	− 1.00 (− 2.00, − 2.00)	
Difference 15 min—12 weeks				
Mean (SD)		− 0.69 (± 0.35)	− 0.05 (± 0.12)	*P* < 0.01**
Median (IQR)		0.00 (0.00, − 1.00)	0.00 (0.00, − 1.00)	

*SCASS* Schiff cold air sensitivity test, *SD* Standard deviation, *IQR* Interquartile range, *MWU Mann–Whitney U test was used, **t test was used. statistically significant at *p* value < 0.05

The performance of the GIC sealant (Ionostar Plus & Easy Glaze, VOCO) was assessed (Table 8) using modified USPHS criteria via the study examiners (xx & xx) in the 12 weeks follow-up. Twenty hypersensitive MIH molars were sealed in the upper jaw and twenty-two molars in the lower jaw, one patient (*n* = 1) was excluded from the analysis due to no-show in a follow-up appointment (Drop-out *n* = 1). A complete loss of the GIC sealing occurred in 3 (7.2%) teeth over 12 weeks. Regarding marginal discoloration without penetration to the pulp, this event was observed in 8 teeth (19.0%) after 12 weeks. Whereas, the majority of the GIC sealant (*n* = 34, 80.9%) retained a shiny surface texture and only 3 (7.2%) GIC sealant had a rough surface texture. Furthermore, the sealing deteriorations occurred mainly in three domains, which were marginal integrity, and optical as well as tactile surface texture (Table 8).

**Table 8:** Clinical scores of the restorations in both groups according to the modified USPHS criteria after 12 weeks post application of GIC on hypersensitive MIH molars (tooth level; n = 43; Drop-out, n = 1)

*N* = 43	Baseline			12 weeks			Total sealings evaluated
	A*	B*	C**	A*	B**	C***	
Retention	42 (100%)			24 (57.3%)	14 (33.5%)	3 (7.2%)	42 (97.6%)
Marginal integrity	42 (100%)			19 (45.2%)	23 (54.8%)	7 (16.6%)	
Marginal discoloration	42 (100%)			33 (78.6%)	8 (19.0%)	1 (2.4%)	
Surface texture- optical	42 (100%)			34 (80.9%)	8 (19.0%)	0 (0.0%)	
Surface texture- tactile	42 (100%)			27 (64.2%)	12 (28.6%)	3 (7.1%)	
Caries	42 (100%)			40 (95.2%)	2 (4.8%)	–	

A = Alpha, B = Bravo, C = Charlie

## Discussion

Molar-incisor hypomineralisation (MIH) is a delimited qualitative developmental defect, which is known to be associated with several dental complications like hypersensitivity, increased caries risk, and aesthetic impairment leading to a significant reduction in OHRQoL (Solinas et al. 2021). Up-to-date, several clinical trials have been conducted recently to evaluate the remineralisation and desensitising effect of various products e.g. fluoride varnish, casein-amorphous calcium phosphate (CPP-ACP), hydroxyapatite containing and arginine containing toothpastes in addition to silver diamine modified atraumatic restorative technique (SMART) and sealant application (Bakkal et al. 2017; Biondi et al. 2017; Restrepo et al. 2016). Though the management of hypersensitivity is still a huge challenge in daily practice (Rapaso et al.,^2019^). Thus, this clinical prospective study provides more insight and evidence on the management and the extend of hypersensitivity reduction in MIH molars with and without enamel breakdown (MIH TNI- 3 & 4).

This study involved 25 patients with a mean age of 8.6 (± 1.85) years, which is slightly higher than those of a similar previous study as shown in Table 1 (Bekes et al. 2022). This might be explained by the fact that this study included not only newly erupted hypersensitive MIH molars but also patients with persisting hypersensitivity after prior desensitising treatments and one adolescent patient with 2nd permanent molar. The presence of enamel hypomineralisation on the second permanent molar is not unusual, this can be supported by the finding of a previous study, which concluded that the occurrence of enamel hypomineralization in the permanent second molar is generally more prevalent when the permanent first molar exhibits molar incisor hypomineralization (MIH) especially with severe defect (De Farias et al., 2021).

The assessment of hypersensitivity is known to be subjective, which is difficult to measure. Evaporative stimuli (Schiff air cold sensitivity score) and visual analogue scale (VAS) are the most popular methods to induce and measure pain intensity in dentine hypersensitivity (Bijur et al. 2001). Since the pain intensity in the air test is higher the than tactile test, the Schiff air cold sensitivity score (SCASS) test was used as the primary outcome parameter in this study (Bekes et al. 2017). However, the additional subjective assessment of hypersensitivity was done using the Wong-baker faces scale (WBFS) instead of the visual analogue scale (VAS). This was decided as WBFS was originally created for children to communicate pain through facial expressions (Facial scales) and the most preferred way for children to report pain (Wong & Baker, 1998; Keck et al. 1996). Furthermore, this study included children with a history of previous desensitising treatments, but no desensitising treatment should have been performed within 4 weeks. This can be explained by the assumption that a minimum period of 2–4 weeks “wash-out phase” is required to eliminate or at least reduce the desensitising effect of a previously applied treatment (Vano et al. 2018).

According to baseline characteristics of the study sample, a slightly higher number of hypersensitive lower molars were observed (*n* = 20, 46.5% upper quadrants vs. *n* = 23, 53.5% lower quadrants). This is similar to the observed characteristics of hypersensitive MIH-affected molars in a previous study (Bekes et al. 2017), whereas the slight majority of the included hypersensitive MIH molars were of high grade of sensitivity SCASS 3 (*n* = 24, 55.8%) as shown in Table 3. This supports the conclusion of previous findings, that hypersensitivity in MIH molars is significantly affected by the quality of mineralisation and degree of hypomineralisation rather than the size of the MIH defect (Linner et al. 2021). Hence, in this study hypersensitive MIH molars with and without enamel breakdown (MIH-TNI 3 & 4a/b) were included.

Regarding the degree of hypersensitivity and its pain intensity, the results of this study showed a mean SCASS of 2.56 (± 0.50) and a mean WBFS of 5.86 (± 2.50). This is similar to a previous study, which involved a mean SCASS of 2.4 (± 0.50) but a higher mean of WBFS 7.1 (± 1.7) as depicted in Table 3 & 4 (Bekes et al 2022). According to a previous epidemiological study, MIH molars of high intensity of hypersensitivity are mostly represented by maximum scores of SCASS (Raposo et al. 2019).

This clinical trial was able to report a hypersensitivity relief, which is evident by a significant decrease (*P* < *0.001, paired t test****)*** in the SCASS score, which reduced from 2.56 (± 0.50) to 1.14 (± 0.96) immediately after 15 min and even further to 0.71 (± 0.89) in the following 12 weeks period as shown in Table 4. A similar significant results were observed with a prolonged use of 8% arginine and calcium carbonate paste over 8 weeks, in which SCASS scores dropped from 2.1 (± 0.3) to 0.8 (± 0.9) (Bekes et al. 2017). Moreover, two other previous studies reported the use of an experimental paste based on zinc-hydroxyapatite which was shown to be effective in reducing hypersensitivity in MIH molars as well. They concluded the non-inferiority of a toothpaste containing microcrystalline hydroxyapatite compared to amino fluoride containing toothpaste (Ehlers et al. 2021; Butera et al. 2023). However, the major problem of hydroxyapatite-containing toothpaste is the lack of fluoride, which is known to be essential for caries prevention (AAPD, 2023). Importantly, this study involves assessing the effect of a single application of GIC sealant (Ionostar Plus + Easy Glaze, VOCO). Hence an assessment of immediate effect (15 min after application) was not only possible but showed a clear clinical benefit.

In this study, for the majority of MIH molars the hypersensitivity SCASS score were reduced to ≤ 1 immediately (15 min post application; 65.1%) and to a SCASS score of 0 over a period of 12 weeks (52.4%, Table 3). This means that the majority of patients (*n* = 34, 80.9%) didn’t show pain or hypersensitivity to the air stimulus anymore. This can be verified through the subjective assessment of pain intensity via WBFS, which showed a significant reduction in the mean WBFS score (*P* < *0.01, paired t test, *Table 4*)* from 5.86 (± 2.50) to immediately after the application of 3.00 (± 2.28) and after 3 months to 2.95 (± 2.17). The result of this study is in a way different than those of a previous study, which aimed to assess hypersensitivity relief of 2 different sealing techniques (resin-based sealant & GIC-based sealant) in different times point. It showed a drastic decrease of the mean SCASS from 2.3 (± 2.17) at baseline to 0.4 (± 0.7) immediately after the treatment and to 0.1 (± 0.41) after a time span of 12 week. Whereas, the results from a randomised control trial (RCT aimed to assess hypersensitivity reduction of silver-modified atraumatic restorative technique SMART (SDF + GIC), which showed a significant reduction from 1.77 (± 0.83) to 0.08 (± 0.28) for SMART, in which SMART showed a slightly more reduction in MIH hypersensitivity than SDF (Balikaya et al. 2022). This difference in the end-point SCASS score may be explained by the fact that the baseline SCASS in this study was initially higher than those of the two mentioned studies. Moreso MIH molars with persistent hypersensitivity referring to teeth which were in a way unreceptive to received previous desensitising treatments were also included (n = 13) (Bekes et al. 2022).

A recent study showed an improved oral health-related quality of life (OHRQol) in children with hypersensitive MIH molars after receiving an application of sealant coverage (Bekes et al. 2021). Thus a sealant coverage might be a helpful and simple tool to improve OHRQoL, due to the fact that a higher severity of MIH-TNI correlated significantly with impaired OHRQoL (Sekundo et al. 2024).

In this study, hypersensitive MIH molars of a baseline SCASS score of 2 & 3 reported a statistically significant reduction of hypersensitivity immediately and in 12 weeks period (P < *0.05*, Mann–Whitney U test). Though still some of the hypersensitive MIH molars with baseline 2 (*n* = 2, 10.5%) and SCASS 3 (*n* = 13, 54%) reported a persistent hypersensitivity (SCASS ≥ 2) as shown in Table 5. Based on a recent systematic review, due to difficulty in assessing subjective pain intensity, which is affected by the inherently subjective and intricate nature of pain, the clinical management of hypersensitivity is mainly defined as a contemporary approach through utilising a combination of in-office application adjacent with home-based desensitising agents such as toothpaste containing potassium salt, fluoride and hydroxyapatite (Dionysopoulos et al. 2023).

However, the effect is more prominent after a 12 weeks period, in which hypersensitive MIH molars with a baseline SCASS of 2 showed a statistically higher reduction in the mean SCASS than those of baseline SCASS 3 (*P* = 0.03, *t* test***;*** Table 5). This difference can be explained by the fact that all hypersensitive MIH molars with baseline SCASS of 2 included in the study showed no post-treatment hypersensitivity (SCASS < 2) while some of the MIH molars with high intensity of hypersensitivity (SCASS 3; *n* = 8, 34.8%) reported a persistent hypersensitivity (Table 5). Based on previous studies, in almost all the patients, who received desensitisation treatments independent of the material used, an improvement in the SCASS values was observed., This emphasizes the hypothesis of starting from very high sensitivity values and a decrease over time. Repeating or combining desensitisors and enhancing sensory changes will stabilise the situation with time (Murri Dello Diago et al. 2021).

In addition, the study analysis involved assessing the effect of GIC sealant application (Ionostar Plus + Easy Glaze, VOCO) on hypersensitivity reduction of MIH molars with (*n* = 13, 30%) and without a history of desensitizing treatment (*n* = 30, 70%) as shown in Table 6. Furthermore, this study included hypersensitive MIH molars with at least no history of desensitizing treatment prior to 1 month before the GIC sealant application. Previous studies investigating various treatment modalities on MIH hypersensitivity showed a significant end-point desensitizing effect within 4 weeks (Bekes et al 2022, 2017). Nevertheless, the study results didn’t show any statistically significant difference in hypersensitivity reduction in MIH molars with and without prior desensitizing treatment (*p* < 0.05). Whereas, hypersensitive MIH molars with no history of pre-treatment showed a higher rate of post-GIC-sealant hypersensitivity (SCASS ≥ 2; *n* = 9, 40.9%) than those without a history of pre-treatments (SCASS ≥ 2; *n* = 3, 23.1%) after 12 weeks post GIC sealant application (Table 6). Interestingly in this study, the pre-treatment group showed a statistically significant reduction in desensitization in the time frame of 15min – 12 weeks (*p* < 0.05). This supports the fact that the frequency of desensitisation treatment on MIH molars is also important for the longevity of the desensitising effect and that this technique is effective irrespective of the type of pre-treatment (Balikkaya et al., 2022).

One secondary aim of the study involved the evaluation of the retention rate of GIC (Ionostar Plus + Easy Glaze, VOCO) on hypersensitive MIH molars over a period of 12 weeks. Based on the study results, fourteen GIC sealants (33.5%) were completely lost over a period of 12 weeks. In two of these cases there were even the presence of dentine caries (4.8%, Table 7). This retention rate is slightly lower than those of a similar study, which observed partial loss of the GIC sealant in 8 teeth (21.1%) but no caries presence over a period of 12 weeks (Bekes et al. 2022). This lower performance rate of the GIC sealant in this study might be explained by the fact that the study involved MIH molars with enamel breakdown to some extent more than the surface coverage of a conventional GIC sealant, which could be still used as an initial quick treatment until definitive restoration is placed (Fragelli et al., 2015). However, both studies showed comparable domains of deteriorations, which were marginal integrity and tactile surface texture (Bekes et al. 2022).

According to a retrospective analysis of the retention of resin-based sealant on MIH molars, the retreatment rate was higher within 2 years and the failure risk was three times more than molars without MIH (Kotsanos et al. 2005). Based on the findings of 4-years follow-up study, a significant enhancement in the retention of fissure sealants in hypomineralised molars was observed when a single bottle adhesive system was applied prior to the placement of the resin-based sealant (Lygidakis et al. 2009). Whereas according to another clinical study, loss of the retention of resin-based sealant in MIH molars was observed in three teeth out of 25 teeth within four weeks (Fragelli et al., 2017), this is lower than those of the present study, in which three out of 42 GIC sealant were lost after 12 weeks.

The fact that hypomineralised enamel has a higher porosity, reduced degree of hardness and elasticity with a change in carbon-carbonate ratios when compared to sound enamel (Elhennawy et al., 2017). This results in compromised bonding due to poor adhesion and reduced bonding strength in MIH-affected enamel (Lagarde et al. 2020). Thus the application of GIC sealant might be superior to resin-based sealants, which require optimal conditions such as excellent moisture control (Cvikl et al. 2018). On the contrary, glass ionomer cement sealants can serve as a suitable temporary treatment option in challenging clinical conditions where isolation is insufficient. This is due to their lower technique sensitive and their ability to set quickly without the need for intermediate steps like etching. This is particularly advantageous in cases where a sealing is necessary for a not fully erupted MIH molar, as well as for non-cooperative, anxious children or patients experiencing hypersensitivity and pain in their molars. The straightforward application procedure of glass ionomer cement sealants makes them a more convenient choice for children. Additionally, these sealants may offer the added benefit of fluoride release, making them a viable option for tooth preservation and caries protection (Elhennawy & Schwendicke 2016). It has been proposed that the use of a GIC sealant can provide protection for molars affected by MIH against post-eruption enamel breakdown (Lygidakis 2010). The prevailing theory suggests that the fluoride found in this material may permeate into the affected enamel and dentin, promoting mineralization of the hypomineralized areas (Lygidakis 2010).

The present study has some limitations, which included a higher sample size that would have been required for some secondary outcome variables, like the assessment of the retention rate of GIC sealant. A longer follow-up time frame would have been good to evaluate the domains of the deterioration and hypersensitivity relief over a longer period. In addition, the lack of untreated control, which would have been interesting for the interpretation of the study results. However, due to the high degree of hypersensitivity intensity, the lack of control (no in-office treatment) might have been not only unethical but also not feasible as parents would have refused in study participation. A comparative control group involving other standard desensitisation treatments, such as resin-based sealants, CPP-ACP pastes, sodium fluoride (NaF) varnish would have added more value to the results. Further investigations are required to assess hypersensitivity relief of combining GIC sealant with other standard desensitisation treatments such as CPP-ACP pastes, and sodium fluoride (NaF) varnish.

## Conclusion

GIC coverage is highly effective in providing instant relief from hypersensitivity in MIH molars in schoolchildren and the effect even continues over a period of 12 weeks minimum. Furthermore, GIC sealant not only provides a significant reduction in hypersensitivity of freshly erupted MIH molars but also in cases of persistent hypersensitivity.

## Supplementary Information

Below is the link to the electronic supplementary material.

Supplementary file1 (DOCX 33 KB)

Supplementary file2 (PDF 877 KB)

## Data Availability

The dataset used in this study contains sensitive details of paediatric patients, which can’t be uploaded to an open repository for ethical reasons.
